# Modeling Radioimmune Response—Current Status and Perspectives

**DOI:** 10.3389/fonc.2021.647272

**Published:** 2021-03-16

**Authors:** Thomas Friedrich, Nicholas Henthorn, Marco Durante

**Affiliations:** ^1^ Biophysics Department, GSI Helmholtz Center for Heavy Ion Research, Darmstadt, Germany; ^2^ Division of Cancer Sciences, School of Medical Sciences, Faculty of Biology, Medicine and Health, The University of Manchester, Manchester, United Kingdom; ^3^ The Christie NHS Foundation Trust, Manchester Academic Health Science Centre, Manchester, United Kingdom; ^4^ Institute for Solid State Physics, Technical University Darmstadt, Darmstadt, Germany

**Keywords:** radiation immunity, immunotherapy, radiation therapy, modeling, radiation effect

## Abstract

The combination of immune therapy with radiation offers an exciting and promising treatment modality in cancer therapy. It has been hypothesized that radiation induces damage signals within the tumor, making it more detectable for the immune system. In combination with inhibiting immune checkpoints an effective anti-tumor immune response may be established. This inversion from tumor immune evasion raises numerous questions to be solved to support an effective clinical implementation: These include the optimum immune drug and radiation dose time courses, the amount of damage and associated doses required to stimulate an immune response, and the impact of lymphocyte status and dynamics. Biophysical modeling can offer unique insights, providing quantitative information addressing these factors and highlighting mechanisms of action. In this work we review the existing modeling approaches of combined ‘radioimmune’ response, as well as associated fields of study. We propose modeling attempts that appear relevant for an effective and predictive model. We emphasize the importance of the time course of drug and dose delivery in view to the time course of the triggered biological processes. Special attention is also paid to the dose distribution to circulating blood lymphocytes and the effect this has on immune competence.

## Introduction

Supporting immune therapy with radiotherapy is a promising approach in particular to tackle non immunogenic tumors, where formation of distant metastases is one of the main reason for failure of curative therapy (1–3). An important variant of immune therapy focuses on immune checkpoint inhibitors, through appropriate antibodies, to reverse immune evasion within tumors. Radiation can enhance this process if delivered in combination. We shall term such combination therapy “radioimmunotherapy” (RIT) throughout this review article. The underlying paradigm is that the radiation induced damage gives rise to the expression of immune stimulating damage markers such as calreticulin, HMGB1 or ATP, or causes a release of interferon by pathways such as cGAS/STING (4). These processes allow tumor cells to be recognized by their specific antigens and leads to attraction of antigen presenting cells that initiate the activation of effector cells that can eventually inactivate the tumor. The particular role of radiation is therefore thought to restore the visibility of the tumor to the immune system, while the immune therapy antibodies efficiently attack the (now visible) tumor, as well as metastases throughout the body. Indeed, an abscopal effect, i.e. the shrinking or definite cure of metastatic lesions has been observed in situations where immune therapy or radiation therapy alone is likely to fail (5, 6).

The interplay of radiation with the immune system is remarkably versatile and has been readily acknowledged as one of the key aspects of radiation therapy (7): In RIT, radiation offers a systemic therapeutic potential, while classical radiotherapy acts targeted and restricted to the tumor region only. The interaction of radiation induced damage with the immune system is even visible when radiation alone is given: On the one hand, even when given alone radiation at high doses can act immunostimulating by supporting the activation of antigen presenting cells (8) and by increasing T-cell infiltration and the expression of MHC class 1 exploited for antigen presentation (9). However, at the same time the radiation action can suppress the immune system, e.g. by irradiation of draining lymph nodes, inhibiting effector cell activation (10–12). This eventually results in lymphopenia, which is known as a common side effect of radiation therapy (13–17) and associated with a worse prognosis. Likewise, radiation may cause upregulation of immune checkpoints, paving the way for a durable escape of the tumor from immune surveillance (18). Hence, from a mechanistic point of view, it is not clear under which circumstances radiation can really be supportive for immune therapy, and quantification of processes and effects are needed to approach this challenge.

Currently, RIT is used in several hundred clinical studies, and more than 50 of those are already in phase 3 (9). The investigations mostly focus on melanoma, non small cell lung cancer, head and neck squamous cell carcinoma and breast cancer. There are a number of FDA approved drugs that focus on inhibiting the immune checkpoints relevant for the lymphocyte activation (CTLA-4) and lymphocyte effects to the tumor (PD-1 or PD-L1). Current clinical strategy is to convert non-immunogenic ‘cold’ tumors into immunogenic, ‘hot’ ones, which allow the infiltration and action of immune effector cells, mostly CD8+ T-lymphocytes. Also here, the question for the optimal therapeutic setting arises, and a quantification of the optimum doses and schedules as well as of success rates are urgently needed.

This challenge is approached by radiobiological modeling, and the aim of the present work is to briefly review modeling approaches associated with aspects of RIT as well as to show potential future developments of this field of research. We thereby also update recent review articles (19, 20) that are partially concerned with the state of the art in modeling RIT as well.

## Model Approaches

In the context of RIT some modeling approaches have been developed that either aim at simulating the outcome of such therapy, or to model specific underlying processes that impose a rationale for RIT. In particular, five major model approaches describe RIT using checkpoint blockers. Other models focus on the immunomodulatory effect of either radiation or checkpoint blockers alone, without considering their combination. A further class of model simulates in detail pathway related aspects on the way to establish the radioimmune response.

### Models for Radioimmunotherapy

At present (February 2021) to the knowledge of the authors there are only five consistent model approaches that attempt to simulate the full course of RIT published in peer reviewed journals. Typically, the models consist of a number of coupled differential or difference equations, each of which describes the dynamics of a key quantity such as the amount of lymphocytes or tumor cells. They differ in mathematical structure, level of detail, type of checkpoint blocker(s), and type of dose and concentration response function. **Table 1** gives an overview of the present models.

**Table 1:** Models for RIT and general properties.

Publication	Blocked checkpoint(s)	Number of interacting quantities
**Serre (** 21 **)**	PD-1, CTLA-4	3
**Chakwizira (** 22 **)**	IDO (in context of glioblastoma therapy)	3
**Poleszczuk (** 23 **)**	Not specified, but applied to CTLA-4	4
**Kosinsky (** 24 **)**	PD-1	5
**Byun (** 25 **)**	PD-1, PD-L1	4

The models address different checkpoint blockers, as given in the second column. They establish the interactions of tumor cells, immune cells, and eventually signals such as antigens via coupled dynamic equations, and the third column indicates the number of these quantities and equations. As PD-1 and PD-L1 form an axis, models applicable to PD-1 blocking are applicable to PD-L1 blocking as well.

To describe the effects of RIT, Serre et al published the first encompassing model framework (21). In this pioneering work the authors modeled the immune system by the amount of effector cells, whose amount is determined by the expression level of tumor antigens. While radiation causes the expression of such antigens, aPD-1 is giving clearance for the effector cells to shrink the tumor mass. The authors attributed the role of aCTLA4 to the long term immune response, i.e. the memory effect, while neglecting its role in fostering effector cell activation even at early times after irradiation. Serre et al. formulated their model as a set of coupled time dependent difference equations. The dose of checkpoint blockers results in quite specifically chosen nonlinear relationships into tumor cell removal, while radiation is assumed to lead to tumor cell inactivation according to the well accepted linear quadratic formalism. The synergism between radiation and checkpoint blockers establishes via the radiation stimulated antigen expression that promotes the immune response, if checkpoints are blocked. Serre et al. solved their set of equations numerically and could describe nicely tumor growth curves of a preclinical experiment. In a later publication (26) they added more dynamical information by allowing time for the antigen release and immune response to establish. As those time scales may interfere with radiation fractionation schemes they introduced the concept of immunologically effective dose (IED), which is the counterpart of the biologically effective dose (BED). BED and IED are the total radiation doses that would be delivered in infinitely many fractions to result in the same targeted and abscopal effect, respectively, as for a regimen with a given fractionation scheme.

Chakwizira et al. (22) simplified Serre’s original model to explain the immune response of radiation in combination with a checkpoint blocker targeting IDO, which is used in the context of treating glioblastoma. They only considered the short time immune response and replaced the complicated response after PD-1 checkpoint blocking in Serre’s model by a simplified dose response to IDO blockers. They succeeded to reproduce with their model experimentally determined survival times of rats with glioblastoma for different radiation doses alone or in combination with immune therapy. Using the model prospectively they predicted that hypofractionation without unusual long gaps (> 1d) maximizes the synergy between radiation and immune therapy.

Poleszczuk et al. (23) developed a model approach including simple, linear interaction terms reflecting immune cells affecting the tumor. They also include a continuously time delayed removal of tumor cells that are committed to death, and distinguish between immunogenic or radiation induced death. To simulate abscopal effects, they rely on their prior work for effector cell motion, explaining variations in transport to distant metastatic sites. Accelerated primary tumor growth appears in favor of abscopal effects due to detraction of immune cells. Interestingly, in that framework they predict a worse prognosis for treating a primary tumor in case an abscopal tumor is present, as the latter one would attract effector cells as well and thus stands in competition with the primary one.

In the model presented by Kosinsky et al. (24) a logistic tumor growth is modified by radiation essentially following the LQ formalism and by the presence of T cells. Here, the latter is amplified, triggered by cell death via an enhancement of the immune activation rate that depends on the PD-1 checkpoint blocker. While being similar to Serre’s model, this approach is more versatile as both undifferentiated and differentiated T cell compartments in the tumor microenvironment are simulated, and there is an explicit dynamic formulation for the removal of dying cells. However, this is established at the expense of numerous parameters, which makes the model harder to validate. Nevertheless, the authors managed to mostly reproduce experimental findings in giving aPD-1 concomitantly or subsequently to radiation therapy.

The model of Byun et al. (25) provides an explicit simulation of both the PD-1 and the PD-L1 concentration, which mainly determine the interaction between tumor cells and T cells. They assume a decaying action strength of both radiation and administered drugs, and consider the binding kinetics of immune checkpoints, modified by checkpoint blocking antibodies. Their model is benchmarked at hand of one rich data set. The model properties are also investigated by a sensitivity analysis and by systematically inspecting model predictions depending on input parameters.

### Models for the Immune Response After Either Irradiation or Checkpoint Blocking Alone

In the literature also a number of models can be found that consider the immunomodulatory action of radiation or of checkpoint blockers alone, without considering their combined action. Such a rather isolated consideration may be useful to inspect the individual agent based effects before considering their combination, which is expected to be more complicated due to additional synergistic mechanisms. A selection of models is compiled in **Table 2** and summarized below.

**Table 2:** Selected models for immune response after radiation or immune therapy with checkpoint blockers alone.

Publication	Considered agent
**Alfonso (** 27 **)**	radiation
**Valentinuzzi (** 28 **)**	aPD-1, aPD-L1
**Lai and Frieman (** 29 **)**	aPD-1
**Milberg (** 30 **)**	aPD-1 and a-CTLA-4
**Radunskaya (** 31 **)**	aPD-L1
**Nikolopoulu (** 32 **)**	aPD-1
**Butner (** 33 **)**	Any checkpoint blocker
**Wilkie (** 34 **)**	Unspecified

Alfonso et al. (27) suggested a model for the immune modulatory effect of fractionated radiation. It starts off from a quite detailed model of tumor growth dynamics, accounting for hypoxic avascular and potentially necrotic regions inside the tumor. The level of available effector cells in the tumor microenvironment is promoted by radiation, which is modeled via a reaction kinetics approach. The degree of effector cell infiltration is modeled empirically as well, and they account for a delayed shrinking of the tumor mass after irradiation. Their model suggests that the level of functional vascularization is an important determinant for therapy design rather than tumor size alone.

The work of Valentinuzzi (28) implemented Gompertzian tumor growth model and explicitly simulated modes of inhibition of the PD-1 – PD-L1 axis. They furthermore distinguish between unaffected tumor cells and tumor cells that are continuously being removed after immune cell attack.

Lai and Frieman (29) modeled the interaction of a tumor, several types of immune cells, DAMP release and PD-1 checkpoint blockers on a quite detailed level, including active transport and/or diffusion of these quantities. They also modeled vaccinations with drugs promoting tumor infiltration of T cells, and their model supports the belief that infiltration is a necessary precondition for successful immunotherapy.

Milberg et al. (30) used a physiologically based pharmacokinetic model for deriving the impact of checkpoint inhibition. They follow a quantitative systems pharmacology approach, describing the dynamics of many involved factors explicitly.

In the model of Radunskaya et al. (31) the authors investigated the immune cell dynamics within the spleen, the blood and the tumor. Modeling interaction between those compartments, their model allows to calculate the impact on tumor growth, and blocking of PD-L1 modifies these interactions.

The work of Nikolopoulo et al. (32) considers in particular the dynamics of PD-1. They present a stability analysis of the underlying equations in the case of no therapy, where they find an equilibrium between T cells and cancer cells. Including checkpoint blocking antibodies they present also a sensitivity analysis.

Butner et al. (33) presented a model approach that intends to describe the clinical outcome of immune therapy. Remarkably, they applied their model to clinical data and demonstrated its capability for discriminating between therapy responders and non-responders based on early assessments of tumor growth. The model uses methods of statistical physics, where the transport of drugs and cytokines are described by diffusion. They finally derive an approximate, but fairly simple formula, which is used for further evaluations.

Wilkie et al. (34) instead employed a logistic tumor growth model, which is modified by a predation mechanism reflecting immune cells, that themselves are promoted by the presence of tumor cells. They used their model to explain the phenomenon of transient tumor dormancy. Although the authors did not employ a specific mechanism for checkpoint blocking, the interaction function has been set up quite generally and can be easily gauged to contain such immune suppressing factors.

### Models at the Level of Underlying Pathways

Modeling of immune responses at the pathway scale becomes rather difficult due to the complexity of the underlying protein networks, which are usually not completely known. Understanding at the pathway scale is, however, desirable for a number of reasons, for instance to identify mechanistic steps that can be targeted through intervention. Although the mechanisms are often understood qualitatively, the quantitative data required for model construction tends to be missing, mostly due to a lack of experimental accessibility. However, some models at this scale do exist, despite lack of data and resultant uncertainties, albeit primarily focused on modeling the immune response alone, discarding interactions with radiation.

Gregg et al. introduced a systems biology approach model of DNA sensing and interferon production (35), later expanding the model to describe the cGAS-STING pathway (36). The model describes the dynamics of the pathway through a series of “states”, with ordinary differential equations determining transitions between states and enzyme reactions described through Michaelis Menten kinetics. In total the model uses 13 states giving rise to 34 model parameters; 13 describing cGAS, 11 describing JAK/STAT, and 10 describing degradation rate parameters (e.g. DNA degradation by TREX1). The model parameters are fit to experimental data where possible and unknown parameters are optimized (25 unknown in total, with all cGAS related parameters assumed to be unknown). The model calculates molecule concentrations within the cytosol as a function of time; including cGAS, STING, DNA, IFNβ, and TREX1. The authors are able to reproduce findings such as drug inhibition of cGAS described through mass action kinetics. The detailed modeling approach allows for investigation of more potent drugs through the variation of association constant. The authors also performed a sensitivity analysis of their model and showed that IFNβ activity is highly robust to perturbations in TREX1 feedback, making the model less dependent on the particular choice of the corresponding input parameters. In the same way, the insensitivity of IFNβ production on TREX1 activity also provides a testable hypothesis. Whilst the model presented by Gregg et al. (36) was not specifically designed for the combined radiation action it is not difficult to foresee modifications that would accommodate this; for example, modifying the initial amount of cytosolic DNA as a function of radiation quality and dose.

The extent, and success, of an anti-tumor immune response is dependent on immune cell activation and signaling. To that end a number of mathematical models have been designed to probe these mechanisms. For example, Mesecke et al. (37) developed a mathematical model of natural killer (NK) cell activating/inhibitory signal receptors at the molecular level. The model is designed with “optional” modules to investigate mechanisms, giving rise to 72 individual models of NK activation and inhibition. Similarly, a number of mechanistic models have been developed to describe T-cell activation (38, 39).

Immune response modeling spans many scales, from the molecular level up to the patient level (40). Palsson et al. (41) developed a framework to integrate published subset models into a single multiscale model through parameterization, known as the Fully-integrated Immune Response Model (FIRM). In FIRM connectivity matrices are built between subset models to describe the global network structure. The model specifies antigen exposure over time and calculates antibody levels and cell concentrations. Although initially designed to simulate the immune response to tuberculosis infection FIRM is also capable of simulating the cellular response to tumor challenge.

Beyond these models, a number of tools are available to simplify and increase the accessibility of immune process modeling. Such tools as BioNetGen (42), Cell studio (43), NetLogo (44), and Simmune (45) may be helpful in simulating various aspects in a pathway oriented modeling of radioimmune response.

## State of the Art in Modeling Radioimmunotherapy

To summarize, there are numerous model approaches that cover aspects of RIT. These approaches can be distinguished, as used to structure the present review, by their level of applicability: Some models focus on specific pathways or on the general interaction between a tumor and the immune system, other approaches model the immunologic response to checkpoint blockers or radiation alone, and a few models even attempt to model RIT. On all these levels, models are found with different levels of detail.

As RIT approaches a standard treatment for some cancer types, such modeling is needed to interpret clinical outcome in a quantitative way. However, there are various gaps in our knowledge on biologic aspects of the immune response, and therefore these models accumulate open parameters. Furthermore, due to a lack of clinical experience a validation and benchmarking can only occur with preclinical experiments, where usually growth dynamics of implanted synthetic tumors has been investigated.

Comparing the five models for RIT summarized in **Table 1**, one finds a number of similarities. All models describe the dynamics of cancer cells, immune cells and eventually of signals leading to recruiting activated T cells (DAMPs, antigens, IFN release, e.g.) via rate equations, i.e. coupled differential or difference equations. They all include that dying cancer cells finally provoke lymphocyte presence, which is why radiation can trigger this process. Checkpoint blocking is included in all models as a key to admit lymphocyte action. It is worth to note that the complicated network of a variety of different immune cell types and multiple underlying processes like different modes of immune mediated cell inactivation is not reflected by any model. Models are rather kept simplified, working with effective quantities e.g. by ignoring different types of lymphocytes. For instance, most models do not distinguish between helper and cytotoxic T cells, because finally the amount of effector cells of whatever type are of interest, while other cell types may support their presence as mediators and are simulated implicitly, i.e. without explicitly appearing in equations. This level of abstraction is for a good reason: A corresponding model to such detail would require a plethora of model parameters, thereby ruling out robust predictions.

**Figure 1:**
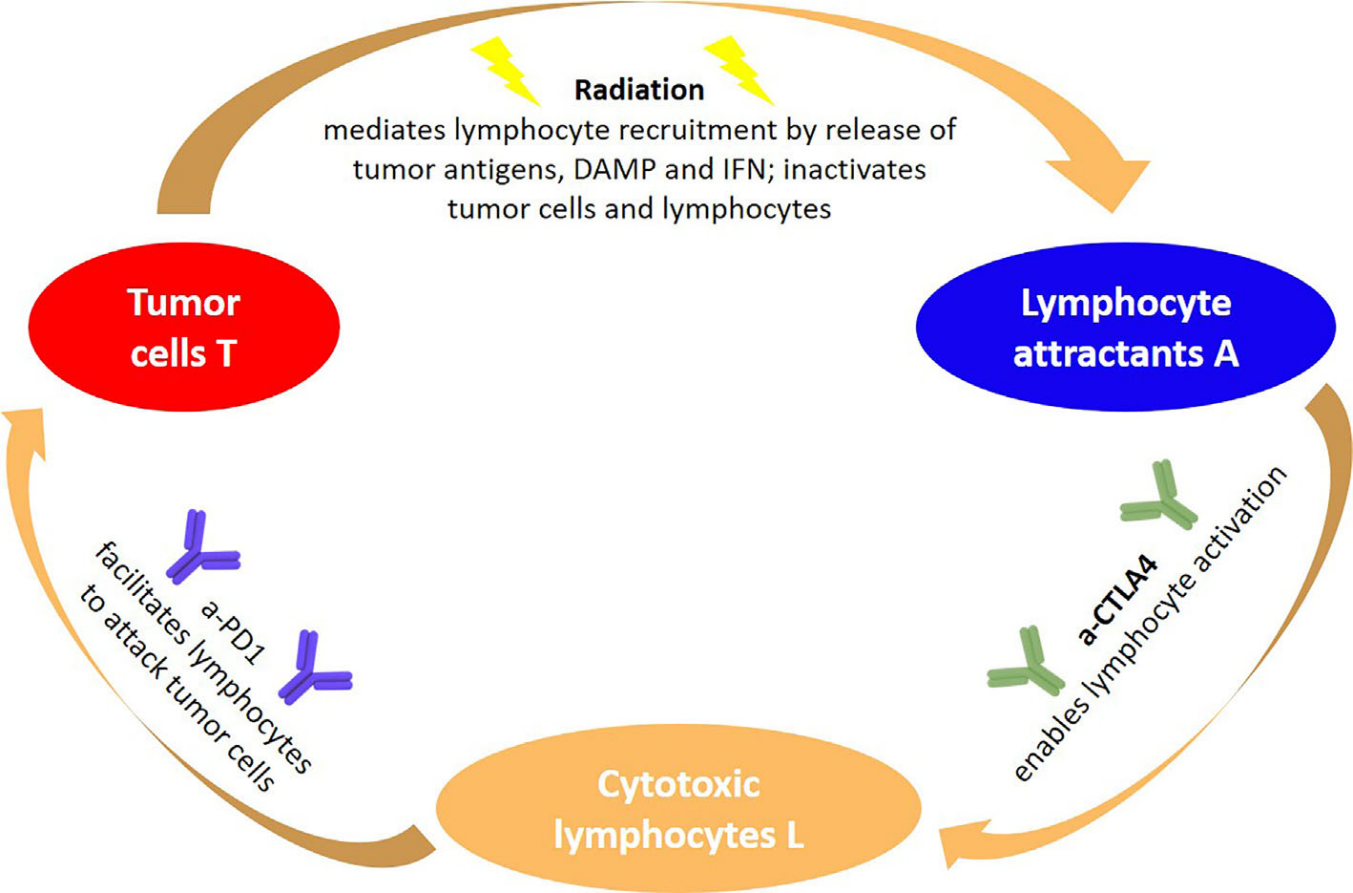
General paradigm underlying RIT using immune checkpoint blockers from a modeler**’**s perspective: The abundance of tumor cells, lymphocyte attracting signals and activated lymphocytes are three quantities that depend on each other, but are also impacted by external agents such as radiation and immune checkpoint blockers. The synergy of coupling radiation and immune therapy emerges, as radiation amplifies signals that are exploited for tumor cell recognition, which in combination with aCTLA-4 lead to an effective lymphocyte activation, resulting in a tumor cell predation driven by cytotoxic lymphocytes.

The underlying paradigm including aCTLA-4 and aPD-1 as checkpoint blockers are visualized in **Figure 1** in a modeler’s perspective, identifying three key quantities and three agents. The sequence of processes reflects the immunity cycle as suggested by Chen and Mellmann (46), where here radiation is the primary cause of cell death of tumor cells, which at the same time triggers a systemic antitumor response. With some modifications, a similar circle of dependencies could be established for the IDO checkpoint instead of the PD-1 and CTLA-4 checkpoints. Notably, there are many more molecules involved in the immune checkpoints than the ones the checkpoints are named after, and the expression of these molecules is a dynamic process that may be modified also by other agents than radiation, e.g. heat (47). Again, models tend here to simplify the situation and model effectively the onset of immune activation triggered by radiation induced cell death.

**Figure 2:**
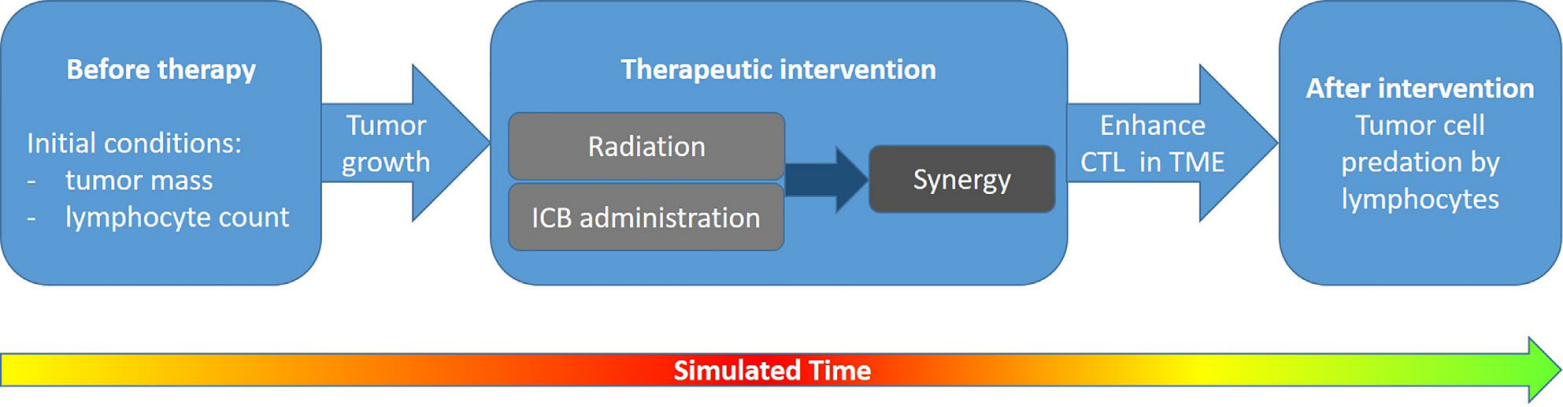
Logic underlying the simulation of combined radiation and immunotherapy effects: In a first step, initial conditions are defined which characterize tumor growth and the immune system**’**s capability (e.g., represented by the number of lymphocytes effectively taking part in tumor cell eradication) without therapy. In a second step the targeted radiation effects as well as checkpoint blocking is simulated, leading to a synergistic immune response. Finally, this results in an enhancement of cytotoxic T lymphocytes (CTL) in the tumor microenvironment (TME) of the irradiated and—if applicable—of an abscopal site. The T cells may eradicate tumor cells, which eventually leads to tumor control. If the therapy design is successful, typically within the therapy block or shortly after the tumor growth will turn into shrinkage as indicated by the color scale in the time arrow (green, no tumor; red, large tumor mass).

As a further similarity, all models follow the general idea of simulating the dynamics of T cells at the tumor site(s), eventually capable to predate tumor cells. This is performed by first defining initial conditions and then following tumor growth and its possible turnover into shrinkage by the synergism of radiation exposure and immunotherapy, as indicated in **Figure 2**.

The models, however, differ in many aspects as well: They use various tumor growth models without considering radiation or immune effects, which might be reasoned partially in the experimental data used for benchmarking. They differ further in the particular choice of interaction terms between immune effector cells and tumor cells. They also vary in the selection of subclasses of tumor cells (hypoxic, inactivated but not yet removed…) and immune cells (CD4+, CD8+, dendritic cells, …) employed. Needless to say, as they are complex models they differ in the number of open parameters (i.e. degrees of freedom of the model), the knowledge about experimentally inspired fixed model parameters, and the associated numerical values. Model limitations are directly connected to the particular choice of modeled cell types, interaction processes and functional dependences and need to be investigated for each model separately.

As the models are established from authors with somewhat different background and perspective, they all use a different terminology and a different notation, making comparisons in view of their complexity quite tedious. Finally, they are validated against experimental data to different levels, while a comprehensive validation across multiple data sets has not been demonstrated so far at all. Nevertheless, all models managed to recover the experimentally observed amplification of immune response by radiation and allow investigation of impacting factors such as dosage, fractionation scheme and the drug administration schedule.

## Future Perspectives

Rather than only considering fully integrated models for predicting the outcome of RIT, the approaches at a lower level of applicability may be important to test and maybe reject underlying assumptions in comparison with experimental data. We would like to stress that also model approaches that attempt to model general functionality of the immune system or the interaction of a growing tumor with the immune system provoked many model based investigations (48–53), that may help to optimize model strategies.

Several differences between the models have been pointed out above. Although these may seem technical and a matter of proper implementation at the first glance, they may have a strong impact on the simulation outcome. These include the choice of the tumor growth model as well as the functional relationship expressed by the interaction terms. Here various assumptions need to be tested against available data, e.g., there is no unique answer on how many activated cytotoxic T-cells are needed to effectively remove a single tumor cell, and how tumor cells can be accessed by T cells due to space limitations.

Besides overcoming such open questions in current models, the following key questions appear to be most promising to be addressed by the following future model approaches, which shall be briefly discussed below:

- What does the therapy response to radiation and checkpoint blockers look like, and how can underlying *response times* be used to optimize scheduling?
- How do primary tumor and abscopal sites differ in *availability and accessibility of T cells*?
- What is the role of radiation induced *lymphocyte inactivation* in cases where the radiation field covers a large portion of vascularized tissue or lymph nodes?
- What is the potential role of *high LET radiation* in regard of the previous aspects?

*Response times:* A very important aspect that is rarely discussed is the time delay between involved biological steps. For instance, cells killed by radiation are not killed immediately. Rather cell death is a process, and therefore also DAMP signals will be elicited with some delay after irradiation. Likewise, the removal of cells will take place later, and T cell activation and recruitment also takes time. In some approaches tumor cells that are inactivated will be removed according to an ordinary decay differential equation, leading to an exponential removal. However, it would be much more plausible to introduce peaked distribution functions with a support on a finite time interval (i.e. with a maximum value) for such delay times.

*Availability and accessibility of T cells*: Concerning the attempt to model the immune response in an abscopal tumor that is not irradiated at all, the crucial question is, what amount of radiation amplified T cells will migrate to this tumor site. It is unclear to what extent other draining lymph nodes except the one corresponding to the irradiation site contribute to the pool of activated lymphocytes. While models at the moment can only use assumptions, future dedicated experimental studies could provide more insight. Such studies might help to decide whether activated T cells in the microenvironment of the primary tumor, the abscopal tumor and in the blood need to be modeled separately, eventually including spatial aspects, or can be simply related, thereby reducing the number of degrees of freedom.

*Lymphocyte inactivation:* Another aspect not sufficiently included in models so far is suppression of the lymphocyte status by radiation. While models account for such suppression eventually in the tumor region, the lymphocyte pool can be largely inactivated, or, if lymph nodes are in the radiation field, the number of naïve T cells can be reduced so that a replenishment is strongly inhibited. This idea demonstrates that radiation has both an immune stimulating and immunosuppressive effect, and modeling could help to determine optimum doses, investigating the impact of dose rate and treatment modality etc. Indeed it is evident that patients with lymphopenia have a worse therapy prognosis (12–17). Experimental studies indicate that (i) lymph node irradiation is a crucial factor that should be avoided, if possible (10, 11), and that lymphocytes in general are quite radiosensitive and hence the blood pool is vulnerable by radiation (54–60). There are only few studies on the dose response of radiation induced lymphopenia (61–63), considering the dose distribution within the blood pool. Again, general models of lymphocyte dynamics (64) may contribute to further model developments.

*High LET radiation:* The role of proton and carbon ion therapy in the context of RIT is at the moment rather unexplored but appears to be quite promising (65, 66): With protons and carbon ions a very conformal irradiation of the target is achieved. In particular, for carbon ions, additional biological advantages result in an enhanced effect in the target region and allow for hypofractionated regimens (67, 68). In the context of RIT this promises at least a twofold advantage: First, sparing normal tissue allows for a tremendous reduction of effective field sizes and allows for less reduction of the lymphocyte pool. With an appropriate field design, lymph nodes could even be spared as well (66). If however lymph nodes are affected by metastases, they may be specifically treated by elective node irradiation using small treatment fields, although the benefit of such treatment remains debated in conventional therapy (69–71). With sufficiently conformal fields, surrounding organs at risk, but also circulatory lymphocytes and unaffected nodes can be spared. The precise conformal irradiation can be realized using ion beams (72, 73). Second, with carbon ions large doses as frequently applied in RIT can be generically realized with comparably tolerable side effects to the normal tissue. This is ultimately reasoned in the high LET effects to cells and tissues, i.e. providing a large relative biological effectiveness and overcoming the resistance of cells in S/G2 phases or of hypoxic cells in the target region. One may conjecture that the damage complexity inflicted by high LET radiation gives rise to a third advantage: Overcoming the radioresistance of hypoxic cells and in general locally clustered DNA damage could lead to a larger level of immune stimulation. One may suspect about a more efficient activation of the cGAS/STING pathway or an enhanced release of DAMPs (74, 75). Considering the temporal pattern of the immune response, the time scales between irradiation and radiation effects are expected to be modified after high LET radiation, accounting for the more severe inflicted damage (76, 77). A faster manifestation of cell inactivation as compared to low-LET radiation suggests a more rapid immune activation and T cell recruitment, while at the same time providing a stronger delay in tumor growth. On the other hand, the immunosuppressive effects of radiation as PD-L1 upregulation may be modified and eventually amplified by the enormous energy concentration within high LET ion tracks. Also at the moment it is not clear whether or not high LET radiation will enhance the infiltration capability of T cells in the tumor microenvironment, or whether the latter will be modified in other aspects. First experimental results (78–80) do not show a clear picture yet, while the tissue sparing effect of particle irradiation indeed seems to be beneficial regarding lymphocyte deprivation (81). Thus the multiple perspectives and open questions associated with the use of high LET RIT warrant further preclinical experiments, which are able to answer the speculative and encouraging expectations presented above.

From the considerations regarding high LET radiation one might also expect that hypofractionated irradiation of small fields would be most suitable for RIT. For SBRT regimens, e.g. enough cells would be inactivated in the target area to set on the immune stimulating effect, while in the small entrance channels only a smaller fraction of lymphocytes in the blood stream would be affected (82). Indeed, early clinical experience using SBRT in combination with checkpoint blockers indicates therapeutic benefits (83).

The four radiobiological questions that have been identified and discussed above can be addressed both experimentally and theoretically. For theoretic model formulation it is an important aspect that the considered experimental data are comprehensive, i.e. self-consistent data sets where multiple observables (e.g. CD 8+ cell count, DAMP release and tumor masses) are simultaneously analyzed for various (many) treatment conditions. Such data sets are most profitable, as they can be directly used for comprehensive model gauging. This approach is complementary to applying models to multiple, independent data sets which is a rather convenient strategy for model testing, thereby supporting or falsifying the underlying mechanistic assumptions. More comprehensive data sets will therefore potentially allow to better assess distinct model approaches. For simplistic models the task is then to choose the most important key quantities that determine tumor mass dynamics, and for very detailed models following an ab-initio approach implementing OMICS data in immune response models (84) may help to keep model uncertainties comparably low despite a high number of degrees of freedom.

Generally, to support the models’ validity and to test their assumptions a broader benchmarking against experimental data is desirable. A fruitful strategy would be to apply one model with fixed model constants (except those characterizing a particular experiments) to multiple independent data sets. At the moment there exist quite a number of thoroughly analyzed experimental data sets (mainly tumor growth dynamics) of primary and abscopal tumors with various doses and fractionation schemes etc., e.g. (4, 10, 85–89). In that line, theoretic models may come up with specific predictions, to interpolate between the results, and identify interesting treatment scenarios to be investigated. Experiments will be able to answer these questions, thereby making our current understanding of the combined action of radiation and immunotherapy more precise. We would like to stress here, that there is still a lack of apparently basic experiments such as investigating interferon release in dependence of radiation quality and dose. Such systematic quantification experiments, although not directly related to current ‘hot topics’, would be very valuable to establish a consistent mechanistic understanding of interactions between radiation and the immune system. Also, experiments are not available where both radiation dose and checkpoint blocker drug concentration are varied systematically. Such experiments would be very valuable to quantify the expected synergism of radiation and checkpoint blocking. Generally, a dialogue of modelers, immunologists and oncologists will be needed to decide about the necessities and options to go for, aiming for a quantification of radio immune response.

Concerning the use in the clinics, the pivotal role of RIT modeling is to indicate factors to account for in treatment planning. This includes, for instance, the need to consider the entire vascular system and the blood pool as an organ at risk for lymphopenia (90). Degrees of freedom that need to be optimized are the radiation dose, the fractionation schedule, radiation type, geometry of the irradiation field(s), irradiation angles, antibody type, drug concentration and drug delivery schedule. This also includes exhibiting the role of emerging radiation therapy modalities such as FLASH or spatial fractionation, which may also spare the blood pool efficiently, but for which the interaction with the immune system is unclear at the moment (91).

Within therapy, the adaptive change or adjustment of initial treatment strategies may be aided or reasoned by model approaches. The advantage of mathematical models as compared to general perceptions is that they are quantitative in nature. At this moment models are quite successful in describing preclinical experiments. In near future, an emerging task will be to develop corresponding model description tailored to clinical situations. For integrated modeling in clinical practice, eventually involved in treatment planning, the step from preclinical experiments towards application in therapy of patients has to be thoroughly validated. In this regard, experience of RIT in patients will be of particular importance, and existing models may be supportive in finding interpretations that help to improve schedules and dosage, and in the same way eventually experience gradual validation.

Thus, benchmark data for such models will be generated within ongoing clinical studies, and hopefully models will acquire predictive power that finally can be used in decision making and treatment planning.

## Author Contributions

TF and NH designed and wrote the manuscript. MD contributed with input on the general outline and the potential capabilities of high LET radiation. All authors edited and proofread the manuscript. All authors contributed to the article and approved the submitted version.

## Funding

NH is supported by the European Union’s Horizon 2020 research and innovation program under grant agreement No. 730983 (INSPIRE).

## Conflict of Interest

The authors declare that the research was conducted in the absence of any commercial or financial relationships that could be construed as a potential conflict of interest.
